# Identification of imaging-based pulmonary and extrapulmonary treatable traits in COPD: a review

**DOI:** 10.3389/fmed.2026.1852728

**Published:** 2026-07-22

**Authors:** Xingbo Wang, Yuhan Peng, Yuke Hu, Tao Zhu

**Affiliations:** 1 School of Medical and Life Sciences, Chengdu University of Traditional Chinese Medicine, Chengdu, China; 2 College of Medical Technology, Chengdu University of Traditional Chinese Medicine, Chengdu, China; 3 Respiratory Medicine and Critical Care Medicine, Suining Central Hospital, Suining, Sichuan, China

**Keywords:** airway lesions, chronic obstructive pulmonary disease, emphysema, medical imaging, treatable traits

## Abstract

Chronic obstructive pulmonary disease (COPD) is characterized by marked pathological heterogeneity, and traditional pulmonary function tests fail to support precise individualized therapy. Imaging-defined treatable traits have emerged as a novel strategy for COPD precision management. This review comprehensively describes the imaging manifestations, quantitative metrics and clinical implications of treatable traits across COPD lung parenchyma, airways, pulmonary vessels and major comorbidities, with a focus on CT and MRI techniques. According to existing clinical evidence, imaging biomarkers and matched treatments are divided into three tiers. A number of validated imaging indicators can effectively guide stratified interventions, including pulmonary rehabilitation, medication, bronchoscopic intervention and surgery. Nevertheless, the clinical translation of imaging assessment is hindered by non-unified quantitative standards, technical limitations of advanced imaging equipment, insufficient prospective evidence, and the inability to differentiate reversible and irreversible lesions. Further efforts are required to standardize imaging protocols, carry out prospective clinical trials, optimize multi-modal imaging and artificial intelligence tools, and improve evaluation systems for COPD comorbidities. In conclusion, imaging-based treatable traits have great potential to refine COPD diagnosis and treatment, and further high-quality research is needed to facilitate its widespread clinical application.

## 1 Introduction

Chronic obstructive pulmonary disease (COPD) is a highly heterogeneous chronic inflammatory disease characterized by persistent airflow limitation and structural alterations of the lung parenchyma, airways, and vasculature (1). Affecting over 392 million people worldwide and accounting for approximately 3.3 million deaths annually, COPD is the third leading cause of death globally. The high morbidity and mortality impose a substantial social and economic burden. Despite its high prevalence, COPD remains underdiagnosed, with many patients not being diagnosed until the disease has reached an advanced clinical stage (1). Early diagnosis is of critical clinical value for delaying disease progression, optimizing treatment strategies, and improving patient outcomes. Although pulmonary function tests (PFTs) serve as the gold standard for the clinical diagnosis of COPD, their accessibility and operational complexity remain major limitations (2). In recent years, with the emergence of the “treatable traits” (TTs) concept in COPD management, imaging techniques have evolved from auxiliary diagnostic tools into core components of precision medicine, providing unprecedented structural and functional information to guide individualized treatment decisions (3).

TTs refer to pathophysiological phenomena that can be modified by specific interventions (e.g., pharmacotherapy, surgery), including parenchymal destruction, airway remodeling, pulmonary vascular abnormalities, and comorbidities. Using high-resolution CT and quantitative CT analysis, clinicians can precisely quantify structural abnormalities such as emphysema, airway wall thickening, and pulmonary artery dilation (e.g., PA/AO ratio > 1.07). These features are not only associated with disease severity but also serve as key indicators for predicting treatment response and guiding individualized therapy (4, 5).

With the rapid development of radiomics and artificial intelligence, imaging assessment of COPD is moving toward a more precise and personalized era. By extracting and analyzing multi-dimensional features from CT/MRI images and integrating them with clinical data (e.g., symptoms, exacerbation history, biomarkers), predictive models can be constructed to identify patient subgroups that are responsive to specific treatment regimens (e.g., anti-inflammatory drugs, bronchodilators, or surgical interventions). However, the clinical translation of imaging techniques still faces numerous challenges, including standardization of parameters, model generalizability, device accessibility, and the integration of imaging with clinical data (4, 6, 7).

**Figure 1:**
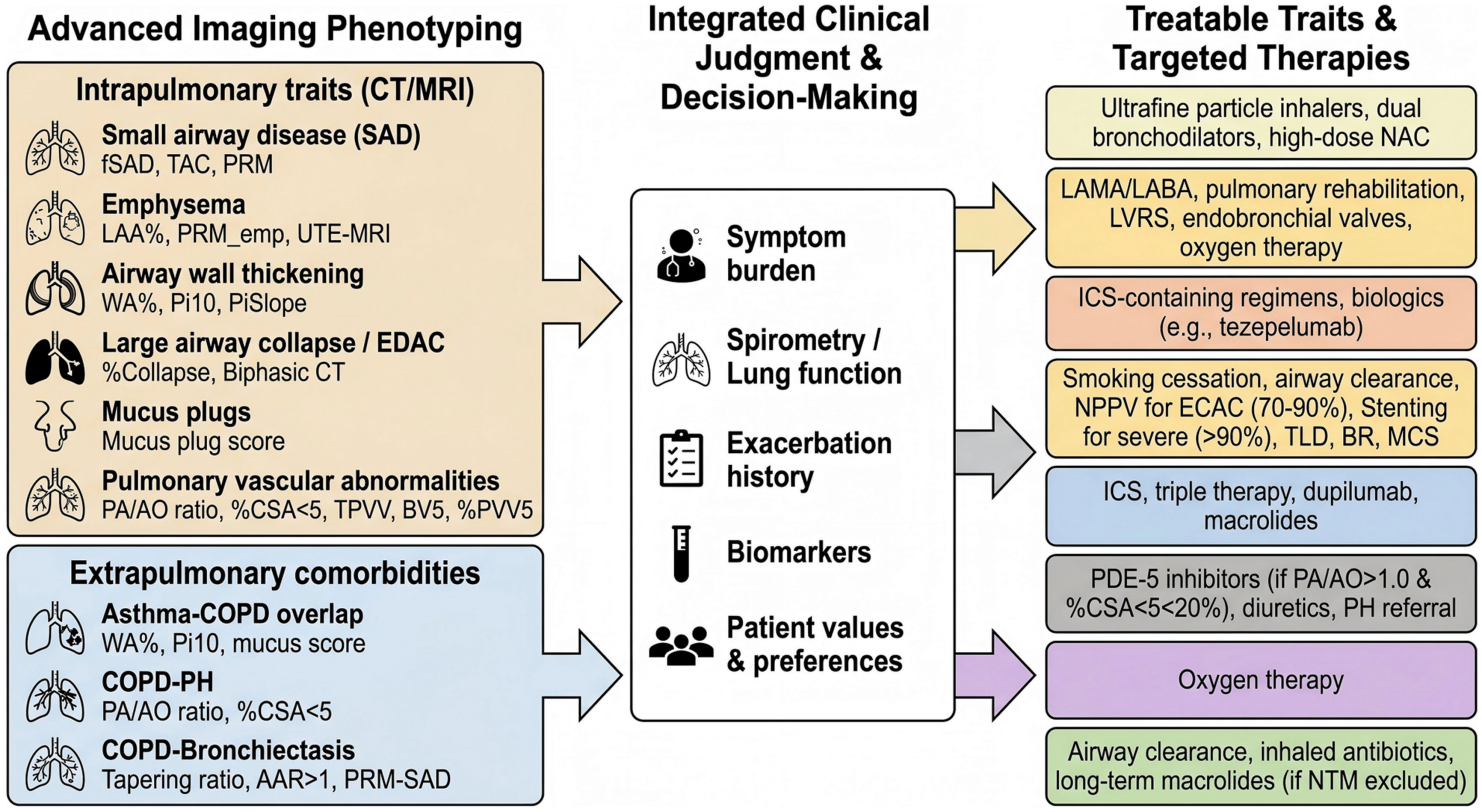
Integrated framework of imaging-defined treatable traits in COPD. The figure illustrates the decision-making pathway from COPD heterogeneity to targeted therapies via advanced imaging phenotyping (including intrapulmonary traits and extrapulmonary comorbidities) combined with integrated clinical judgment. Solid arrows indicate established imaging-guided therapies, dashed arrows indicate imaging-informed adjunctive therapies, and dotted arrows indicate emerging or investigational therapies. Detailed evidence grading is provided in Table 1.

This review aims to systematically explore the identification of treatable traits in COPD imaging assessment and their association with treatment decisions, with a particular focus on the value of CT and MRI in evaluating the lung parenchyma, airways, and vasculature. By comprehensively elucidating the relationship between imaging and treatable traits, we hope to provide clinicians and researchers with a framework for achieving more precise COPD management using imaging techniques, thereby enabling early diagnosis and treatment of COPD and improving patients’ quality of life (Figure 1).

## 2 Imaging phenotypes and identification of treatable traits in COPD

Imaging plays a critical role in COPD by effectively presenting underlying pathology and clearly delineating both pulmonary and extrapulmonary structural features, making it an excellent noninvasive assessment method (8).

### 2.1 Imaging phenotypes and identification of treatable traits in airway disease

#### 2.1.1 Imaging recognition and treatable traits construction in large airway disease

Large airway disease, as an important phenotype of COPD, is characterized primarily by airway wall thickening and tracheal stenosis. Among these abnormalities, expiratory central airway collapse (ECAC), particularly the pathological manifestation known as excessive dynamic airway collapse (EDAC), plays a critical role in disease progression and symptom exacerbation (9). EDAC is mainly caused by a reduction in elastic fibers and decreased tension in the posterior membranous wall of the airway, leading to excessive bulging of the posterior wall into the lumen during expiration and resulting in functional airway stenosis (9). This dynamic collapse is closely associated with several underlying pathological mechanisms in COPD, including chronic airway inflammation, long-term hypoxia, repeated pressure loading, and airway remodeling. Furthermore, long-term use of high-dose corticosteroids may also increase the risk of EDAC (10). Although bronchoscopy remains the gold standard for diagnosing EDAC, its invasive nature limits its applicability in some patients, and it is difficult to comprehensively evaluate multi-level airway lesions. In contrast, biphasic dynamic chest CT, owing to its non-invasive nature and high accuracy (89–97%), has become the preferred imaging method for assessing large airway disease (11). Biphasic dynamic chest CT captures airway changes at end-inspiration, end-expiration, and during the expiratory phase, enabling quantification of the degree of collapse (9). Currently, the diagnosis of EDAC is primarily based on the percentage of collapse (%Collapse), defined as the percentage reduction in airway cross-sectional area (CSA) or diameter during expiration relative to inspiration (12). Multiple studies have demonstrated that the degree of collapse in EDAC patients is positively correlated with symptom severity. Specifically, more severe collapse is associated with more pronounced dyspnea; the percentage of collapse is positively correlated with the CAT score, and patients with a higher degree of collapse experience a significantly reduced quality of life (13). Furthermore, the degree of collapse is closely associated with the risk of exacerbations: patients with a higher degree of collapse exhibit more severe sputum retention, a significantly increased risk of infection, and a notably higher frequency of COPD exacerbations (14). Although prospective studies on the percentage of airway collapse remain limited, this metric may serve as a treatable trait for assessing large airway disease in COPD in the future, facilitating more precise identification and quantification of dynamic collapse of the large airways, thereby providing a basis for individualized treatment. Therefore, an individualized treatment decision framework can be established based on CT-derived quantitative metrics and bronchoscopic findings (15–17): Patients with mild collapse (50–70%) may experience symptom improvement with conservative management, including pulmonary rehabilitation and airway clearance techniques. Patients with moderate collapse (70–90%) may require adjunctive therapies such as non-invasive positive pressure ventilation (NPPV). Patients with severe collapse (>90%) may be candidates for invasive interventions, including laser therapy, airway stenting, or surgery.

#### 2.1.2 Imaging recognition and treatable traits construction in small airway disease

Small airway disease (SAD) is a major feature of COPD. SAD manifests as airway wall thickening, fibrosis of the epithelium, lamina propria, smooth muscle, and adventitia, luminal obstruction by mucus, airway remodeling, and loss of terminal bronchioles (18). Small airways have been recognized as the core site of involvement driving disease progression and airflow limitation (19). Although its importance is well established, the fine anatomical structure and insidious nature of early symptoms pose many challenges to its diagnosis (18). How to accurately identify SAD and quantify its severity remains a difficulty in current research. Currently, pulmonary function testing is the most common and convenient clinical method for evaluating SAD. However, pulmonary function indicators used to diagnose SAD, such as FEF25-75%, have high sensitivity but are limited by a wide normal reference range and high individual variability, making it difficult to distinguish early pathological changes from normal physiological variations when used alone (20). Therefore, imaging has gained increasing attention as an important means to identify SAD. Current imaging methods for evaluating SAD include HRCT scanning, which can directly observe centrilobular nodules, linear branching opacities, and mosaic perfusion, and use parametric response mapping to differentiate emphysema-associated air trapping from small airway disease (21). Studies have shown that the mean lung density expiratory-to-inspiratory ratio (MLDE/I) derived from expiratory and inspiratory CT may be an independent predictor of COPD and severity of airflow obstruction, and that PRM and disease probability measures may serve as novel indicators for identifying small airway dysfunction (22, 23). Furthermore, an established imaging biomarker is the total airway count (TAC), calculated as the sum of visible airways from the third to the eighth generation (24). Although TAC primarily serves as a prognostic biomarker-associated with FEV_1_ decline-it has not yet been validated to guide specific treatments.

In addition, advances in MRI are also noteworthy. MRI uses helium-3 or xenon-129 gas distribution to measure regional ventilation defects, providing spatial distribution information of small airway lesions (25). In hyperpolarized magnetic resonance imaging, the signal distribution of hyperpolarized gases provides ventilation images of lung structure, reflecting regional heterogeneity of airflow and ventilation; the ventilation defect percentage and apparent diffusion coefficient (ADC) are commonly used to assess small airway function (26). This method carries no risk of radiation exposure but is extremely demanding in terms of cost and technical requirements. Furthermore, endobronchial optical coherence tomography (EB-OCT) is a novel imaging method that combines an ultrathin bronchoscope with new navigation technology, allowing visualization of high-resolution cross-sectional structures of the airway wall and providing more information on the small airway wall (27). Combining these imaging indicators with pulmonary function parameters and serum biomarkers provides a multi-dimensional, multi-perspective approach for accurate prediction in the assessment of SAD. Similarly, OCT offers diagnostic insights into airway wall layers, but its role in guiding treatment selection remains at the research stage (28). Clinically, the treatment of small airway disease requires a comprehensive decision-making process that integrates pulmonary function tests, imaging findings, and clinical symptoms, rather than relying solely on imaging biomarkers to guide treatment selection (29). Inhaled therapy regimens should be tailored to patient subgroups (30, 31). Although ultrafine particles have been shown to effectively improve small airway function, their impact on imaging parameters has not yet been demonstrated (32, 33). Additionally, oral mucolytics and antioxidants may confer some degree of benefit on airway function. Future prospective studies are needed to validate the role of imaging biomarkers in guiding the treatment of small airway disease.

#### 2.1.3 Imaging recognition and treatable trait construction in airway wall thickening

Airway remodeling is the primary cause of irreversible airflow limitation in COPD, with airway wall thickening representing the most direct morphological manifestation and a core pathological feature of the disease (34). This process involves abnormal proliferation of structural cells in the airway wall, excessive extracellular matrix deposition, and hypertrophy of submucosal glands. These alterations lead to fixed airway narrowing, reduced elastic recoil, and consequently persistent dyspnea (35). Moreover, these structural changes not only affect airway resistance and lung elastic recoil but are also associated with specific inflammatory phenotypes, suggesting their potential as important biomarkers to guide individualized therapy (36).

Nevertheless, the accurate assessment of airway wall thickening and its application in predicting treatable traits face considerable challenges, including standardization of quantitative methods, cross-device consistency, validation of correlations with clinical outcomes, and the development of predictive models for treatment response (37).

CT is currently the gold standard for assessing airway wall thickening in COPD, offering high spatial resolution (down to the submillimeter level), wide accessibility, and rapid acquisition (38).

Pi10 and PiSlope are key CT-derived quantitative indicators for evaluating airway remodeling in COPD. Pi10 represents the square root of the airway wall cross-sectional area when the internal luminal perimeter is 10 mm, reflecting wall thickness at a specific airway level (39). PiSlope, defined as the slope of the linear relationship between airway wall area and internal perimeter, captures the spatial trend of wall thickness across the entire airway tree and serves as a more comprehensive measure of airway remodeling (40). However, although the definitions of Pi10 and PiSlope are clear, considerable methodological heterogeneity exists across studies in their actual calculation, which constitutes a core limitation of their application. Studies have shown that at least ten literature-based computational methods have been used for Pi10 and PiSlope, with significant differences in key parameters. For example, some methods measure only airways of specific sizes (e.g., Patel and Van Tho methods), others restrict measurements to specific airway generations (e.g., Gietema and Park methods), while still others cover a broader range of airway sizes and generations (e.g., Bhatt, Jobst, and Smith methods). This heterogeneity in calculation methods directly leads to “substantial variability” in Pi10 and PiSlope values across studies, making cross-study comparisons and result interpretation extremely difficult, and limiting their potential as standardized biomarkers (41).

Imaging assessment based on airway wall thickening can effectively guide the classification of COPD phenotypes (42):

Type A: No or only mild emphysema, with or without concomitant bronchial wall thickening.

Type E: Significant emphysema without concomitant bronchial wall thickening.

Type M: Both significant emphysema and bronchial wall thickening, often associated with type 2 inflammation and a higher risk of exacerbations.

Airway wall thickening may also be linked to specific airway inflammatory phenotypes (43). Studies have shown that (43) approximately 20–40% of COPD patients exhibit eosinophil-driven type 2 inflammation, which is often accompanied by more severe airway wall thickening, chronic sputum production, wheezing, dyspnea, accelerated lung function decline, and a higher risk of exacerbations. In contrast, approximately 60–80% of COPD patients have a neutrophil-predominant inflammatory phenotype, which is associated with relatively less airway wall thickening but more frequent corticosteroid resistance (44). For patients with marked airway wall thickening and evidence of type 2 inflammation (blood eosinophil count ≥300/μL), adding an ICS to dual bronchodilator therapy may be considered (2). In patients with severe airway wall thickening and frequent exacerbations in the context of type 2 inflammation, IL-5/IL-5R inhibitors may be effective. The phase III MATINEE and METREX trials showed that mepolizumab significantly reduced the annual exacerbation rate in such patients, and that FEV_1_ remained stable or improved in patients with GOLD stage 2/4 disease (45).

#### 2.1.4 Imaging recognition and treatable trait construction in airway mucus plugs

In 2018, Dunican et al. (46) first proposed visual assessment criteria for airway mucus plugs based on chest CT: a mucus plug is defined as a focal opacity that completely occludes the airway lumen, adjacent to a patent lumen, with radiodensity typically lower than that of adjacent blood vessels. Previous studies have demonstrated that the presence of airway mucus plugs in patients with COPD is associated with worse lung function, reduced exercise capacity, poorer quality of life, and increased risks of all-cause mortality and exacerbations (47, 48).

The formation of mucus plugs involves complex pathophysiological mechanisms (49). Under physiological conditions, the thin mucus layer on the airway surface traps inhaled particles and pathogens and is effectively cleared by ciliary beating and coughing (50). In disease states, impaired ion and fluid transport in epithelial cells leads to excessive mucus concentration, dehydration, elevated osmotic pressure, restricted ciliary activity, and reduced mucociliary clearance. Retained mucus gradually forms plaques and plugs, which obstruct airflow and trigger infection and inflammation (51). Moreover, the hypoxic microenvironment distal to the plug favors anaerobic bacterial growth, further exacerbating airway injury and establishing a vicious cycle (51, 52). The effects of mucus plugs vary according to the level of bronchial involvement (53–55): plugs in the large airways mainly cause airflow obstruction, dyspnea, and reflex coughing; in medium-sized bronchi, they may induce local hypoxia and infection; and in the small airways, they often lead to alveolar hypoventilation, resulting in carbon dioxide retention and hypoxemia. In addition, mucus plugs can elicit local inflammatory responses and promote airway remodeling (56). Currently, quantitative assessment of mucus plugs is performed using a scoring system based on bronchopulmonary segmental anatomy, encompassing 20 segments, with a total score ranging from 0 to 20 (0 = no plug in any segment; 20 = plugs present in all segments). This scoring system provides more comprehensive disease information and helps identify COPD subtypes and guide individualized treatment (57). For instance, patients with a high mucus plug burden may be better candidates for mucolytic therapy or bronchoscopic intervention (57, 58).

It should be noted that mucus plugs are not specific to COPD; they can also be detected in healthy individuals and patients with non-COPD lung diseases. A Canadian study reported (59) a detection rate of 43% in individuals aged 63–87 years without a history of lung disease. In the US SPIROMICS cohort, the detection rate was 25% in healthy subjects with a mean age of 58.9 ± 9.5 years (60). In contrast, the detection rate was only 4.5% in the healthy control group of the SARP 3 cohort (mean age 29.5 ± 11.5 years), suggesting that the presence of mucus plugs may be age-related (46). Therefore, large-scale clinical trials are still needed to validate the use of mucus plugs as a TTs for disease diagnosis, prognosis assessment, and treatment decision-making.

Studies have shown that airway mucus plugs are closely associated with chronic bronchitis (61). Patients with chronic bronchitis exhibit chronic mucus hypersecretion, i.e., excessive mucus production by airway mucous glands and goblet cells, clinically manifested as chronic cough and sputum production (62). Interventional pulmonology treatments targeting the chronic bronchitis phenotype of COPD, such as targeted lung denervation (TLD), bronchial rheoplasty (BR), and metered cryospray (MCS), aim to reduce the source of mucus and control hypersecretion, and have already been introduced into clinical practice (63–65).

In the field of asthma, small-sample studies suggest that biologics may improve mucus plugs. A clinical trial involving 116 patients with severe eosinophilic asthma showed that after 1 year of treatment with benralizumab, patients with mucus plugs experienced improvements in annual exacerbation rate, asthma control score, and percent predicted FEV_1_, as well as a trend toward reduced corticosteroid dose (not statistically significant) (66). Another randomized controlled trial in 109 patients with moderate-to-severe type 2 asthma demonstrated that dupilumab significantly reduced fractional exhaled nitric oxide (FeNO), decreased mucus plugs, improved lung function, and enhanced asthma control (67). These findings suggest potential therapeutic value of biologics for mucus plugs in asthma, but direct evidence in COPD is lacking. Furthermore, CT-identified mucus plugs hold promise as imaging biomarkers for evaluating treatment efficacy.

In summary, airway mucus plugs have the potential to serve as imaging biomarkers for interventional therapy in COPD patients with chronic bronchitis, representing a promising TTs. However, their value in diagnosis, prognosis, and treatment guidance still requires validation through large-scale clinical studies.

### 2.2 Imaging phenotypes and identification of treatable traits in emphysema

As a core pathological component of COPD, emphysema is characterized by the abnormal, permanent enlargement of airspaces distal to the terminal bronchioles and the destruction of their walls (68). On CT imaging, it is quantified as regions with an attenuation below −950 HU on inspiratory scans (42, 69). Visual assessment classically distinguishes three main subtypes (42): (1) Centrilobular emphysema, the most common smoking-related form, originated in the center of the secondary pulmonary lobule and typically progressed from small holes to confluent upper-lobe predominant destruction, associated with distal bronchiolar obliteration. (2) Panlobular emphysema commonly presented in the lower lobes of individuals with alpha-1 antitrypsin deficiency, involving the entire lobule diffusely. Then, smoking accelerated its progression and earlier onset. (3) Paraseptal emphysema affected lobules adjacent to pleural surfaces, such as the mediastinal, costal, and interlobar pleura, and was most frequently observed in the upper lobes (70, 71).

Traditional CT assessment of emphysema mainly relies on density threshold methods (e.g., the percentage of pixels below −950 HU) (72). However, new methods have significantly extended this approach. First, three-dimensional volumetric CT and automated segmentation software (e.g., YACTA) enable automated, unsupervised analysis at the whole-lung and lobar levels, calculating lung volume, mean lung density (MLD), and emphysema index (EI). Their reproducibility is better than that of traditional semi-automated tools, and they reduce inter-operator variability (73). Furthermore, voxel-based analysis of emphysema distribution (e.g., dividing the lungs into 10 equal-volume partitions) reveals craniocaudal heterogeneity, which is closely correlated with pulmonary function indices such as DLCO and PaO2 (74). In addition, artificial intelligence, particularly deep learning, is revolutionizing the imaging assessment of emphysema. Convolutional neural networks (CNNs) are especially adept at automatically learning features from images (75). Moreover, deep neural networks (DNNs) can be used for kernel conversion, transforming archived sharp-kernel CT images into images resembling those from soft-tissue kernels, thereby enabling the reuse of historical CT data for reliable quantitative measurements of emphysema, muscle/fat, and coronary artery calcification (76). Computer-aided diagnosis (CAD) schemes have also been developed for classification based on X-ray dark-field imaging, achieving 100% accuracy in distinguishing diseased from healthy lungs and 93% accuracy in distinguishing emphysema from pulmonary fibrosis (77). Despite the emergence of many novel functional imaging techniques, most have not yet been widely adopted, and the lack of standardized acquisition and post-processing protocols, along with concerns regarding radiation exposure or cost, severely limits their comparability and reproducibility. Future efforts should be directed toward promoting technical standardization and accessibility, integrating multimodal imaging to more comprehensively assess structure–function relationships in the lung, and conducting large-scale longitudinal studies to validate the predictive value of imaging biomarkers for treatment response and prognosis (78).

Lung MRI faced technical challenges due to low proton density, rapid signal decay, and artifacts from cardiopulmonary motion (79). Nevertheless, ultrashort echo time (UTE) MRI emerged as a viable method for assessing tissue loss in COPD, enabling quantification of emphysema through an emphysema index (80, 81). Studies found that UTE—based signal intensity measurements showed good reproducibility and could offer utility comparable to quantitative CT (82, 83). Thus, advances in both CT and MRI not only provided more precise biomarkers for quantifying emphysema but also offered potential for the precise treatment of emphysema in COPD.

Density-based thresholds alone are unable to fully characterize emphysema morphology, leading to the development of morphology-based metrics, such as those differentiating bullous from diffuse disease (84). Paired inspiratory-expiratory CT adds functional insight, air trapping measures (e.g., expiratory mean lung density, kurtosis) and PRM analysis allowing separate quantification of emphysema and functional SAD, potentially outperforming inspiratory LAA% in evaluating airflow limitation (85). It is important to note that PRM is a diagnostic and prognostic tool, valuable for phenotyping and predicting decline, rather than a direct predictive biomarker for a specific pharmacological therapy. However, it plays a clear procedural role in guiding lung volume reduction (86). With advances in artificial intelligence (AI), more imaging biomarkers based on deep learning are being developed. For example, using the Vision Transformer (ViT) model, one can effectively extract the distinctive spatial patterns and textural features of emphysema subtypes (87). Medical management of emphysema includes bronchodilators, corticosteroids, and long-term oxygen therapy (1). Interventional approaches, such as lung volume reduction surgery and endobronchial valve placement, also formed a key part of treatment (88). Pre-procedural CT is essential for evaluating emphysema distribution, assessing fissure integrity, and detecting collateral ventilation. These imaging findings guided target region selection and helped predict outcomes for both surgical and bronchoscopic lung volume reduction (73, 89).

### 2.3 Imaging phenotypes and identification of treatable traits in pulmonary vessel abnormalities

Pulmonary vascular abnormalities, including pulmonary vascular remodeling, microvascular rarefaction, and hemodynamic disturbances, are key drivers of COPD disease progression (90, 91). Traditional imaging assessment mainly relies on subjective visual judgment, making it difficult to detect early or occult pulmonary vascular lesions. However, quantitative analysis techniques developed in recent years, such as CT-based pulmonary vascular volume measurement, calculation of the small vessel cross-sectional area (%CSA), and vessel segmentation algorithms, have significantly improved the accuracy of evaluating morphological and functional changes in the pulmonary vasculature (92).

In recent years, with the development of deep learning and image processing techniques, significant breakthroughs have been achieved in the quantitative imaging assessment of pulmonary vasculature in COPD. The core of automated quantification lies in accurate pulmonary vessel segmentation. Early methods, such as traditional algorithms based on Graph-Cuts and variational region-growing, combined with vessel enhancement filters using Hessian matrices and anisotropic diffusion filters, achieved an average sensitivity of 92.9% and an area under the curve (AUC) of 0.972 on the VESSEL12 challenge dataset (93).

Based on these precise segmentation results, a series of clinically meaningful quantitative parameters have been identified. Total pulmonary vessel volume (TPVV) and the percentage of pulmonary vessel volume relative to lung volume are core metrics for quantifying the overall pulmonary vascular bed (94). More importantly, quantitative indices for vessels of different diameters, such as small vessel volume (BV5, i.e., the volume of vessels with a cross-sectional area <5 mm^2^) and its percentage relative to total vessel volume (%PVV5), have been extensively studied (95). For example, in COPD patients, a reduction in small vessel volume (BV5) is considered a direct imaging manifestation of pulmonary vascular remodeling and pruning, and is closely correlated with the severity of emphysema (96).

These precise quantitative parameters demonstrate significant correlations with the core pathophysiological alterations of COPD. Numerous studies have confirmed that indices reflecting pulmonary vessel loss are significantly negatively correlated with indices reflecting emphysema extent (96). In terms of functional assessment, vascular parameters are also closely linked to pulmonary function indices. For instance, the number of fifth-generation vessels is significantly correlated with FEV_1_% predicted and diffusing capacity (DLco) (97). Additionally, IPVV is independently correlated with airway wall thickness (WT), suggesting that pulmonary vascular pathology and airway remodeling may share a common pathological basis (98).

Meanwhile, automatic separation of arteries and veins has also been refined. By constructing a minimum spanning tree and combining vessel geometric features with CT imaging characteristics, automatic classification of arteries and veins can be achieved on non-contrast CT scans of COPD patients (99). Distinguishing pulmonary arteries from pulmonary veins is of great clinical significance for determining the etiology and hemodynamic impact of vascular pathologies. In COPD patients, pathological changes in the pulmonary arteries and pulmonary veins often differ, and their hemodynamic consequences also vary. Pulmonary artery remodeling primarily increases right ventricular afterload, whereas pulmonary vein abnormalities may affect left ventricular filling and the hydrostatic pressure of the pulmonary circulation. Accurately distinguishing between the two using imaging techniques helps to clarify the origin of vascular pathology.

However, the relevant parameters lack validation from clinical prospective data, and further validation is required before they can be better applied in clinical practice.

Although significant progress has been made in imaging biomarkers for assessing pulmonary vasculature, current studies have not yet incorporated these imaging biomarkers into treatment-related decision-making (100). Clinically, treatment selection still requires comprehensive judgment integrating imaging findings and clinical symptoms (101). Long-term oxygen therapy is key to improving prognosis, as it significantly reduces mortality and the frequency of exacerbations (91). In addition, PDE5 inhibitors (e.g., sildenafil) may be effective in a subset of patients, particularly those with a PA/AO ratio > 1.0 and a percentage of cross-sectional area (CSA) < 5 mm^2^ of less than 20% (102).

### 2.4 Imaging phenotypes and identification of treatable traits in major comorbid conditions of COPD

Asthma is a risk factor for COPD, and the clinical features of both asthma and COPD can coexist in some patients (103). In the past, the term asthma-COPD overlap (ACO) was commonly used to describe such patients. However, ACO is often misinterpreted as a distinct disease entity, whereas its pathogenesis and clinical features are highly heterogeneous. Therefore, the descriptive term “asthma + COPD” is now preferred (1). Regardless of whether ACO or asthma + COPD is used, there remains a lack of consensus on its definition, and different studies have adopted varying criteria for this comorbidity (104). Nevertheless, broad commonalities exist, which may be an important reason for the consistency of findings across studies. In line with the GOLD guideline, this review will subsequently use the term asthma + COPD.

For patients with asthma + COPD, imaging reveals several distinctive features. In the COPDGene cohort, asthma + COPD patients typically show more pronounced airway wall thickening and remodeling on CT, quantified by higher WA% and Pi10 (105). Lu et al. retrospectively enrolled 30 asthma + COPD patients who met both the GOLD criteria for COPD and predefined criteria for asthma symptoms, bronchodilator reversibility, and smoking history, and similarly observed marked airway thickening (106). In the NOVELTY cohort, physician-assigned asthma + COPD patients on hyperpolarised xenon MRI, demonstrated an intermediate pattern between asthma and COPD, with ventilation defects, alveolar enlargement and gas-exchange impairment that are less severe than in COPD alone but more pronounced than in asthma alone (107).

A strict COPD and bronchiectasis association (COPD – BE) should be defined by the ROSE criteria (108). However, in most studies, BE in COPD refers to bronchiectasis detected on imaging in patients with confirmed COPD. Among patients with COPD – BE, HRCT is critical. Typical signs included the “signet-ring” sign (broncho-arterial ratio >1.0), loss of normal bronchial tapering (tapering ratio ≥1.1), and airways visible within 1 cm of the pleura (109, 110). Bronchiectasis is often cylindrical, bilateral and lower-lobe predominant, especially in the left lower lobe (111). Tree-in-bud opacities and centrilobular nodules raise suspicion for non-tuberculous mycobacterial infection (112).

The GOLD criteria for COPD, combined with a resting mPAP >20 mmHg, are often used to diagnose COPD-associated pulmonary hypertension (COPD-PH). The 2022 ESC/ERS guidelines introduced PVR > 5 WU together with mPAP to define severe PH in COPD, whereas previous studies mostly relied on mPAP and cardiac index for its definition (113). For patients with COPD – PH, CT findings are dominated by enlargement of the main pulmonary artery (PA/AO ratio ≥1.0, or ≥0.93 for severe PH) (79, 114). In COPD patients with severe PH, an increased %CSA < 5 suggests predominant vascular remodeling; whereas in cohorts characterized by severe emphysema, a decreased %CSA < 5 indicates an emphysema-predominant pattern with distal vascular loss (115, 116). Four-dimensional flow MRI further shows reduced pulmonary artery helicity, suggesting abnormal haemodynamics and right ventricular dysfunction (117). In summary, each comorbidity has a recognizable imaging signature that can be quantified and used for clinical phenotyping.

Identifying imaging features of COPD comorbidities allows clinicians to target specific TTs. However, it is important to note that imaging features are more likely to serve as clues to identify treatable traits rather than directly guiding comorbidity treatment. For example, in patients with asthma + COPD, imaging findings should be interpreted within a broader multimorbidity disease framework rather than as isolated structural abnormalities. Fangal et al. showed that, compared with asthma or COPD alone, asthma + COPD overlap in smokers exhibits distinct yet overlapping physiological, imaging, and transcriptomic features, indicating that this phenotype is not merely an intermediate state or a structural airway thickening variant of the two diseases (105). In this context, CT-derived airway wall thickening and remodeling, including higher WA% and Pi10, are important not only as morphological descriptors but also as components of a more complex overlapping phenotype that may reflect coexisting airway inflammation, mucus-related disease burden, and alterations in functional impairment (105, 118). These findings suggest that asthma + COPD is best understood as a biologically heterogeneous disease state in which imaging can refine phenotyping, but airway-predominant abnormalities have the greatest clinical significance when interpreted in conjunction with physiological and molecular characteristics rather than in isolation. Therefore, for multiple comorbidities, imaging may be most useful for identifying specific patterns (e.g., an airway-predominant pattern) within a broader precision medicine framework, while emphasizing that treatment decisions should still integrate multiple factors including inflammation, clinical presentation, and functional assessment (105, 119, 120).

Overall, asthma + COPD treatment can follow an asthma-oriented pathway: inhaled corticosteroids (ICS) are essential, long-acting beta-agonist monotherapy should be avoided, and triple therapy (ICS/LABA/LAMA) was recommended (1, 121). If type-2 inflammation is present (e.g., eosinophilia), biologics such as dupilumab can reduce airway wall area percentage and mucus plugs. Furthermore, in eosinophilic COPD, Mepolizumab has been considered to improve airway remodeling (122, 123). For those with a high mucus plug burden, after optimizing inhaled therapy, selective antimicrobial strategies (e.g., macrolides) may be considered (124). Mucus plugs can be seen in both COPD and bronchiectasis; imaging detection of mucus plugs allows airway clearance to be used as a treatment (125). A tree-in-bud pattern suspicious for non-tuberculous mycobacteria required prompt microbiological work-up and avoidance of long-term macrolide monotherapy until infection was excluded (112). An exacerbation-prone phenotype, defined by AI-derived bronchiectasis scores (conicity ratio ≥1.1), whole-lung AAR > 1 proportion (e.g., ≥3%), or higher PRM-SAD, supports the use of long-term macrolides after NTM exclusion, and inhaled antibiotics for chronic *Pseudomonas* infection (110, 125). Most treatment-related studies have been conducted in standalone BE cohorts, and the efficacy and safety of the corresponding therapeutic interventions remain to be validated in COPD – BE cohorts. However, the PA/AO ratio is a diagnostic/screening biomarker for PH, not a predictive biomarker for treatment response. The decision to initiate PDE-5 inhibitors requires confirmatory right heart catheterization, as relying on CT alone could lead to inappropriate treatment (126). In a COPD – PH cohort, sildenafil reduces pulmonary vascular resistance and improves quality of life, without significantly affecting gas exchange (127). However, the use of PDE-5 inhibitors in COPD – PH remains controversial, as some patients may experience worsening gas exchange due to V/Q mismatch, and therapy should be guided by specialist evaluation (128). In contrast, an emphysema phenotype (reduced %CSA < 5, high emphysema burden) is managed with emphysema-directed strategies: long-term oxygen only for severe resting hypoxaemia, and lung volume reduction surgery in carefully selected patients (129). Thus, while imaging-defined features help identify clinically meaningful patterns in COPD comorbidities, their integration with physiological, inflammatory, and microbiological assessments-rather than imaging alone—is essential to guide personalized treatment decisions.

## 3 Conclusion

COPD is a highly heterogeneous chronic lung disorder characterized by progressive structural damage to the lung parenchyma, airways, and pulmonary vasculature. The traditional assessment model, which relies solely on pulmonary function testing, focuses exclusively on functional airflow limitation and fails to capture individual pathological phenotypes. This limitation largely accounts for the substantial variability in treatment responses to uniform therapeutic regimens. The concept of identifying TTs through imaging has opened a new avenue for precision medicine in COPD management (Table 1).

**Table 1:** Imaging – defined TTs in COPD: a framework of phenotypes, biomarkers, and therapeutic strategies.

Imaging-defined phenotype	Key imaging biomarkers (role & calculation)	Therapeutic evidence level & strategies
Large airway disease-dominant	1. Biphasic dynamic chest CT (Diagnostic/Phenotyping) Calculation: Collapse percentage (%Collapse) = (Inspiratory CSA – Expiratory CSA)/Inspiratory CSA × 100% Non-invasive assessment with high diagnostic accuracy (89–97%) for large airway dynamic collapse (11). 2. Percentage of Collapse (%Collapse) (Quantitative Assessment/Treatable Trait) Future treatable trait for assessing large airway disease in COPD (12).	Established Imaging-Guided: Mild collapse (50–70%): Conservative management (pulmonary rehabilitation, airway clearance techniques) (15) Imaging-Informed (Adjunctive): Moderate collapse (70–90%): Non-invasive positive pressure ventilation (NPPV) (16) Emerging/Investigational: Severe collapse (>90%): Invasive interventions (laser therapy, airway stenting, surgery) (17)
Small airway disease-dominant	1. PRM for fSAD (Diagnostic/Phenotyping) Calculation: Voxel-wise analysis: fSAD = Exp_ < −856 HU & Insp_ > −950 HU (85, 130). 2. TAC (Diagnostic/Phenotyping) Calculation: Sum of visible airways from 3rd to 8th generations (131, 132). 3. EB-OCT (Diagnostic/Phenotyping) Description: Direct imaging of 7th-9th bronchial generations (133).	Established Imaging-Guided: None for SAD-specific pharmacological therapy. Imaging-Informed (Adjunctive): Preferential use of ultra-fine particle triple-inhaler formulations for co-deposition in small airways (134). Emerging/Investigational: Dual bronchodilators, high-dose NAC (100), tiotropium combined with theophylline (135).
Airway wall thickening	1. WA% (Wall Area Percentage) (Diagnostic/Phenotyping) Calculation: (Wall Area/Total Area) × 100% (136, 137). 2. Pi10 (Prognostic) Calculation: Derived from linear regression: sqrt(WA) = a + b * Pi. Pi10 = a + b * 10 (138, 139). 3. PiSlope (Prognostic/Investigational) Calculation: Slope of the regression line between sqrt (WA) and Pi (40).	Established Imaging-Guided: NA. Imaging-Informed (Adjunctive): Combination regimens containing inhaled corticosteroids (140). Emerging/Investigational: Biologics (e.g., Tezepelumab, Mepolizumab) – evidence derived from asthma or small single-center COPD studies (141, 142).
Airway mucus plugs	1. Mucus plug score (Prognostic, Diagnostic/Phenotyping) Calculation: Number of involved bronchial segments (46, 143).	Established Imaging-Guided: Smoking cessation (significantly attenuates contribution to airflow obstruction) (144). Imaging-Informed (Adjunctive): Airway clearance techniques (physiotherapy) – suggested by imaging detection of mucus plugs (144). Emerging/Investigational: Interventional techniques (TLD, BR, MCS) – evidence from chronic bronchitis, animal models, and small case series (61, 64, 145, 146).
Emphysema-dominant	1. LAA% (Low Attenuation Area) (Diagnostic/Phenotyping) Calculation: Percentage of pixels <−950 HU on inspiratory CT (42, 69, 147). 2. PRM^emph^ (Diagnostic/Phenotyping, Prognostic) Calculation: Component of PRM identifying emphysema vs. fSAD (85). 3. Fissure Integrity (Surgical/Procedural) Description: Used for evaluating eligibility for lung volume reduction (73).	Established Imaging-Guided: Lung volume reduction surgery or endobronchial valve placement (guided by procedural imaging) (89, 148, 149). Imaging-Informed (Adjunctive): Long-acting bronchodilators (LAMA/LABA), pulmonary rehabilitation, long-term oxygen therapy (150). Emerging/Investigational: NA
Pulmonary vascular abnormalities-dominant	Quantitative CT metrics (Diagnostic/Phenotyping) Calculation: Total pulmonary vessel volume (TPVV) (88). Small vessel volume BV5: volume of vessels with cross-sectional area < 5 mm^2^ (89). PVV5: percentage of BV5 relative to total vessel volume (89). PVV: independently correlated with airway wall thickness (WT) (92). Number of 5th generation vessels (91).	Emerging/Investigational: PDE5 inhibitors (e.g., sildenafil): Potential efficacy in a subset of COPD patients, specifically those with PA/AO ratio > 1.0 and %CSA < 5 mm^2^ < 20% (96). Notably: Relevant quantitative parameters currently lack validation from clinical prospective data; further validation is required before better clinical application (94).

Advanced imaging techniques—including high-resolution CT, biphasic dynamic CT, PRM, ultrashort echo time MRI, and hyperpolarized gas MRI—enable *in vivo* quantitative assessment of core pathological changes, such as emphysema subtypes, EDAC of the large airways, functional small airway disease, airway wall thickening, segmental mucus plugs, and pulmonary vascular remodeling. Moreover, imaging can clearly identify characteristic features of common COPD comorbidities, including asthma, bronchiectasis, and pulmonary hypertension.

Based on the evidence reviewed in this article, imaging biomarkers and their corresponding treatment strategies can be categorized into three levels of evidence.

Level 1 comprises indicators with sufficient evidence and mature clinical application. CT-quantified emphysema severity and fissural integrity effectively screen suitable candidates for lung volume reduction surgery. The percentage of airway collapse measured by dynamic CT stratifies EDAC severity and guides sequential interventions, ranging from conservative management (e.g., pulmonary rehabilitation) to invasive endoscopic or surgical procedures.

Level 2 includes indicators that provide reference value and assist in treatment guidance. Quantitative parameters related to airway wall thickening (WA%, Pi10, PiSlope), when combined with type 2 inflammatory biomarkers, can guide the selection of inhaled corticosteroids and targeted biologic agents. CT-assessed mucus plug burden may support the use of mucolytics and airway clearance therapy. Vascular indicators such as the PA/AO ratio, TPVV, and small vessel volume (BV5) can aid in formulating long-term oxygen therapy and PDE5 inhibitor regimens.

Level 3 encompasses novel techniques and biomarkers still in the exploratory stage, such as EB-OCT, TAC, and most artificial intelligence-based quantitative models. Their clinical value remains to be further validated.

Although quantitative imaging assessment of COPD holds promising potential, several shortcomings—all supported by existing research—must be acknowledged.

First, globally unified technical standards for quantitative imaging assessment have not yet been established. Differences in scanner brands, post-processing software, and measurement algorithms may lead to deviations in the same indicator, resulting in insufficient reproducibility and generalizability, thereby limiting multi-center data sharing and clinical adoption.

Second, imaging techniques themselves have inherent limitations. For example, paired inspiratory-expiratory CT increases cumulative radiation exposure and requires good patient cooperation for breath-holding. Hyperpolarized gas MRI and EB-OCT are expensive and technically demanding, making them difficult to implement in primary care settings.

Third, the majority of current studies on the association between imaging features and clinical outcomes are single-center, retrospective observational studies. There is a lack of large-sample, multi-center, prospective cohort studies with long-term follow-up, as well as randomized controlled trials stratified by imaging phenotypes.

Fourth, the generalization capacity of automated artificial intelligence segmentation models remains insufficient, and rigorous external validation is lacking.

Fifth, the assessment system for COPD comorbidities is inadequate. Imaging features of comorbidities such as asthma, bronchiectasis, and interstitial lung disease often overlap and interfere with one another, making it difficult to distinguish the primary treatable traits.

To address these challenges, future research and clinical translation should be carried out in a phased and targeted manner. First, unified industry standards should be established, including standardized scanning protocols and post-processing workflows for CT and MRI, to eliminate systematic errors caused by different devices and algorithms. Subsequently, multi-center, large-sample prospective cohort studies and imaging-stratified randomized controlled trials should be conducted to validate the predictive value of various imaging biomarkers for treatment response and long-term outcomes, and to clarify which patient populations benefit from targeted therapies. Ultimately, a multi-modal integrated assessment system should be developed that incorporates imaging phenotypes, peripheral inflammatory biomarkers, microbiological indicators, and clinical data to construct multi-dimensional predictive models and to attempt to distinguish reversible from irreversible lesions. Concurrently, technological improvements should be pursued to reduce radiation dose and cost of advanced imaging equipment, simplify interpretation workflows, and enhance accessibility in primary care settings.

In summary, the imaging-based treatable traits framework provides an important direction for overcoming the limitations of traditional COPD diagnosis and treatment and for achieving individualized therapy. However, this field is still in a transitional phase of exploration and translation. Only by progressively addressing issues such as the lack of standardization, insufficient clinical evidence, and limited practical applicability can the full value of imaging be realized. This will ultimately lead to the establishment of a complete, standardized, and widely implementable precision management system for COPD based on multi-dimensional treatable traits across the entire disease course.

## 4 Review methodology

This review employs a narrative synthesis approach to integrate evidence on imaging-defined TTs in COPD. Given the broad and heterogeneous nature of the topic (spanning multiple imaging modalities, disease subtypes, and potential therapeutic targets), we performed a structured literature search, without claiming exhaustiveness, to identify relevant studies.

Search Strategy: We searched PubMed, Web of Science, and Embase databases from January 1, 2000, to February 28, 2026 (adjust timeframe accordingly).

Search Terms: The search strategy combined terms for (“Chronic Obstructive Pulmonary Disease” OR “COPD”) AND (“Imaging” OR “Computed Tomography” OR “Magnetic Resonance Imaging”) AND (“Phenotype” OR “Treatable Traits” OR “Biomarkers” OR “Therapy” OR “Management”).

Selection and Prioritization: We prioritized high-quality evidence including randomized controlled trials (RCTs), large longitudinal cohort studies, meta-analyses, and systematic reviews. Narrative reviews and expert consensus were included to provide clinical context for emerging concepts.

## Glossary

AI: artificial intelligence
BDP: beclomethasone dipropionate
BODE: BMI, Obstruction, Dyspnoea and Exercise capacity index
BR: bronchial rheoplasty
CAT: COPD Assessment Test
COPD: chronic obstructive pulmonary disease
COPD – BE: COPD with bronchiectasis
COPD – PH: COPD with pulmonary hypertension
CT: computed tomography
EB – OCT: endobronchial optical coherence tomography
FEV_1_: forced expiratory volume in 1 s
FF: formoterol fumarate
FRI: functional respiratory imaging
fSAD: functional small airway disease
GB: glycopyrronium bromide
GOLD: Global Initiative for Chronic Obstructive Lung Disease
HRCT: high-resolution computed tomography
HU: Hounsfield unit
ICS: inhaled corticosteroids
LABA: long-acting beta-agonist
LAMA: long-acting muscarinic antagonist
LAA%: low attenuation area percentage
MCS: metered cryospray
mMRC: modified Medical Research Council dyspnea scale
MRI: magnetic resonance imaging
NAC: N-acetylcysteine
OCT: optical coherence tomography
PA/AO: pulmonary artery to aorta ratio
PDE-5: phosphodiesterase-5
Pi10: square root of wall area of a hypothetical airway with 10 mm internal perimeter
pMDI: pressurized metered-dose inhaler
PRM: parametric response map
PRM^emph^: PRM emphysema component
RCT: randomized controlled trial
SAD: small airway disease
SWES: synergic whole-lung emphysema score
TAC: total airway count
TLD: targeted lung denervation
TTs: treatable traits
UTE: ultrashort echo time
ViT: vision transformer
WA%: wall area percentage
%CSA < 5: percentage of cross-sectional area of small pulmonary vessels (<5 mm^2^)
